# The Mechanisms of Plant Cell Wall Deconstruction during Enzymatic Hydrolysis

**DOI:** 10.1371/journal.pone.0108313

**Published:** 2014-09-18

**Authors:** Lisbeth G. Thygesen, Emil E. Thybring, Katja S. Johansen, Claus Felby

**Affiliations:** 1 University of Copenhagen, Department of Geosciences and Nature management, Faculty of Science, Frederiksberg C, Denmark; 2 EMPA, Swiss Federal Laboratories for Materials Science and Technology, Applied Wood Materials, Dübendorf, Switzerland; ETH Zürich, Swiss Federal Institute of Technology, Institute for Building Materials, Wood Materials Science, Zürich, Switzerland; 3 Novozymes A/S, Bagsværd, Denmark; UMass, United States of America

## Abstract

Mechanical agitation during enzymatic hydrolysis of insoluble plant biomass at high dry matter contents is indispensable for the initial liquefaction step in biorefining. It is known that particle size reduction is an important part of liquefaction, but the mechanisms involved are poorly understood. Here we put forward a simple model based on mechanical principles capable of capturing the result of the interaction between mechanical forces and cell wall weakening via hydrolysis of glucosidic bonds. This study illustrates that basic material science insights are relevant also within biochemistry, particularly when it comes to up-scaling of processes based on insoluble feed stocks.

## Introduction

Plant derived materials are at the heart of the evolving bioeconomy and utilisation of plant cell walls for polymers and energy is central. A key step is to separate and depolymerise cellulose for subsequent refining. It is vital for the success of any biorefining scheme to have the cell wall polymers released with the least possible inputs of energy, water and additives [1]. One approach is to break down the cell wall matrix by use of lignocellulolytic enzymes. This enzymatic processing cannot take place at economically feasible dry matter contents without simultaneous input of mechanical energy, i.e. mixing [2], [3], but the combined mechanisms responsible for the change in particle sizes seen during cell wall degradation have not been understood or described before. Here we put forward a simple model based on mechanical principles capable of capturing the effect of the interaction between mechanical forces and cell wall weakening via hydrolysis of glucosidic bonds.

The mechanisms responsible for the beneficial effects of mixing and mechanical agitation during enzymatic hydrolysis are at present not well understood. It has been suggested that mixing prevents local build-up of product and thus counteracts possible end-product inhibition and/or water constraint due to solutes [4]–[7]. One can also speculate that mixing increases hydrolysis speed by contributing to relocation of the enzymes from recalcitrant to degradable regions of the substrate [1], [8]–[10].

**Figure 1:**
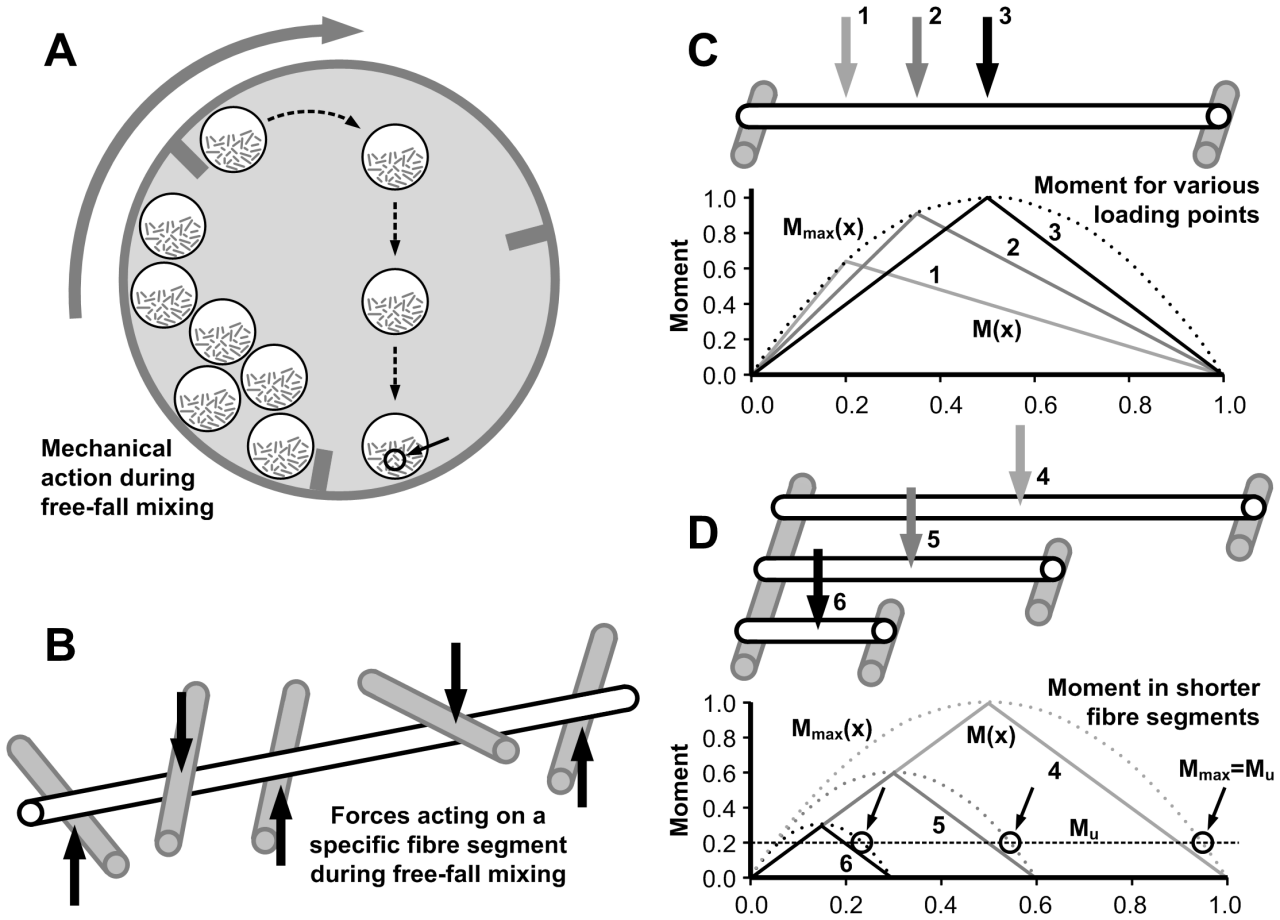
Simulation of fibre attrition during free-fall mixing (A) using beam theory for three-point bending. The mechanical action on fibres (B) is simplified by assuming that fibres only break where generated bending moments (C) are sufficient to cause failure, i.e. where they equal or exceed the failure moment, M_u_ (D). The probable failure region (PFR) extends over the middle 89%, 82%, and 58% of fibres 4, 5, and 6, respectively due to the difference in fibre length between these three fibres.

When biomass is processed by lignocellulytic enzymes fibre attrition is one of the initial effects which lead to a liquefaction of the substrate, as particle size reduction is known to lower viscosity in fibre suspensions [11]. However, the attrition stops or slows down during the later phases of hydrolysis [12]–[14]. A possible mechanical component in the positive effect of mixing could be that forces acting upon fibres make them break in points weakened by enzyme activity. During mixing of a slurry containing elongated thick-walled plant cells such as fibres, mechanical forces from the fibres hitting each other will cause stresses in the fibres, see Figures 1a and 1b. For stiff and elongated cells we suggest to describe the forces at play using simple beam theory for 3-point bending. We further suggest that the probability of fibre breaking is uniform within a certain zone around the centre of the fibre as defined by geometry and material strength. Outside this region fibre breaking will not occur as the stresses generated are insufficient to cause failure. This implies that for a given force, a fibre shorter than a certain threshold will not break, which may help explain the observed stagnation in fibre lengths during the later phases of hydrolysis. Further, we suggest that fibre strength decreases during processing as a consequence of enzymatic activity, thereby decreasing the mechanical force needed to break a fibre.

In this study we compare the actual development in fibre lengths during enzymatic hydrolysis with length distributions generated by a simulation based on these principles.

## Material and Methods

### Enzymatic hydrolysis

**Figure 2:**
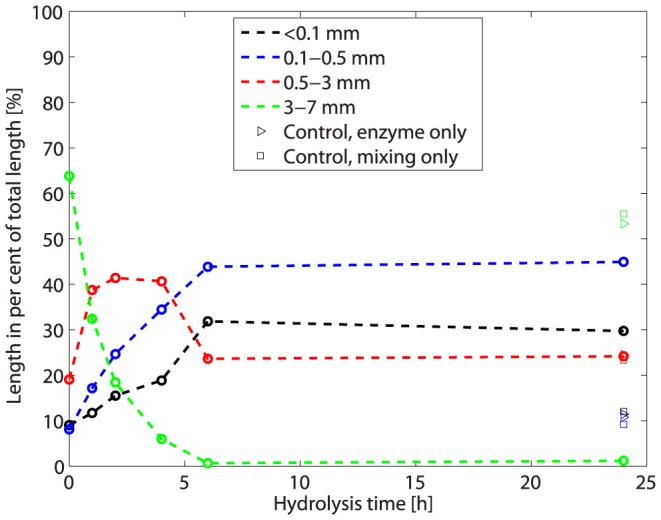
Actual fibre length distributions for flax during enzymatic hydrolysis.

Unbleached flax fibre bundles (*Linum usitatissimum* L.) were purchased from the company Skytten (www.skytten-danmark.dk) and were cut into 3–7 mm segments by use of a razor blade. Hydrolysis was carried out by using a 5∶1 mixture of Celluclast and Novozym 188 at 10 FPU (Filter paper units) per gram dry matter. The enzymes were a gift from Novozymes A/S (Bagsværd, Denmark, http://www.novozymes.com). The enzymes were added to flax together with a 50 mM pH 4.8 sodium citrate buffer. Each sample contained 1 g fibres (dry matter). The dry matter content of the mixture (fibres+buffer+enzymes) was 25%, and the temperature was 50°C. At this high dry matter content all liquid was adsorbed by the substrate at the onset of hydrolysis. The samples were subjected to free fall mixing during the hydrolysis. Free fall mixing is done by using a rotating horizontal mixer. In the present experiment, hydrolysis was carried out in100 mL plastic bottles placed in an 800-mm-diameter horizontal drum rotating at 60 rpm. The drum was equipped with two paddles that lifted and dropped the bottles during rotation. Samples (duplicates) were taken out after 0, 1, 2, 4, 6 and 24 h of hydrolysis. Two different controls were also run in duplicates: (1) free fall mixing only, i.e. the same conditions during hydrolysis as for the time series, but only buffer was added (i.e. no enzymes added); and (2) enzymes only, i.e. the samples were identical to the samples used for the time series, but not subjected to any mixing. Both types of controls were run for 24 h at 50°C. Results for these controls confirmed earlier results [12] and are shown in Figure 2.

Hydrolysis was stopped by boiling the samples in water for 10 min. Finally samples were taken out and for each bottle 0.5 g dry matter was diluted in 0.5 L demineralised water, frozen at −18°C until they were subjected to FiberTester analysis (Lorentzen & Wettre, www.l-w.com) as described in [12]. The FiberTester measures individual particle dimensions using automated image analysis. About 15,000 to 20,000 individual fibres were measured per sample. After FiberTester analysis results for duplicates were pooled, i.e. data for each time point in Figure 2 is based on the dimensions of about 30,000 to 40,000 individual fibres.

### Simulations

All simulations are based on the probable failure region (PFR) described by the part of the fibre length in which the maximum possible generated moment, M_max_ equals or exceeds the strength, i.e. the failure moment, M_u_, see Figure 1c. The worst possible loading situation for a fibre segment, i.e. where M_max_ is largest, is the three-point bending situation where the fibre is supported at the extremes of its length. This loading situation does not necessarily represent the actual loading situation as schematically illustrated in Figure 1b, however, it allows determination of the region of the fibre (PFR) where the generated bending moments are sufficient to break the fibre. In three-point bending the moment distribution is triangular, but the maximum moment generated as loading points are shifted is described by the parabola
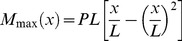
(1)where P is force and x/L is the relative position along the fibre length, L. From this the relative extend of the probable failure region is found as
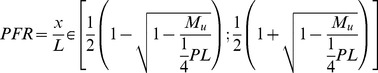
(2)Since three-point bending offers the most extreme loading situation for the fibre, the calculated probable failure region describes where fibre breaking is at all possible given the combination of strength (breaking moment, M_u_) and force (P). Breaking outside of the probable failure region does not occur due to insufficient moments, independent of the actual loading situation.

The forces generated in free-fall mixing can be assumed to be constant over time due to an unchanged overall mass, while the breaking moment most likely decreases over time as a result of hydrolysis. Nonetheless, the size of the probable failure region decreases with decreasing fibre length for constant M_u_ and P as a result of equation (2). This is illustrated in Figure 1d where fibre 6 is one-third as long as fibre 4. While the probable failure region of fibre 4 extends over the middle 89% of the length, it has decreased to 58% for fibre 6, assuming constant M_u_ and P.

Equation (2) was implemented in MatLab R2013a and the free-fall mixing simulated in a step-wise procedure where each fibre in each step is either excluded, is passed on unchanged to the next step or is broken into two new fibres that are forwarded to the next step. First the ratio 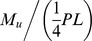, i.e. the ratio of failure moment to maximum moment beneath a central force, see situation 3 in Figure 1c, is found. If this ratio is larger than 1, the fibre is excluded from the simulation because it is too short to break (see equation 2). If the fibre is not excluded, its length is multiplied with a random number between zero and one. If this point falls within the probable failure region, the fibre is cut in two at this position and the two new fibres are forwarded to the next step. If not, the fibre is forwarded unchanged. Thus, a uniform positive probability distribution is assigned to the probable failure region as a result of the random loading situations occurring during free-fall mixing. In each step, M_u_ is reduced in order to simulate the successive weakening of the fibres during hydrolysis. The initial fibre population consists of the actual fibre lengths from the experiment described above (33,518 fibres). The simulation stops when less than 100 fibres are forwarded to the next step. Simulations were run several times, but results were practically indistinguishable, and in this presentation only results for single runs are shown.

## Results and Discussion

It was possible to simulate the actual development in fibre lengths during enzymatic hydrolysis of flax fibres, including the stagnation in fibre length decrease observed after a certain processing time [12]–[14]. This result indicates that particle size development during enzymatic degradation of insoluble plant materials can be understood if the mechanical principles involved are combined with the biochemical hydrolysis.

The actual distribution of flax fibre lengths during hydrolysis is shown in figure 2. A fibre length reduction is seen within the first 6 hours, where the longest fibres (3–7 mm) disappear, while the medium length fibres (0.5–3 mm) increase their share within the first 1–2 hours and then decrease to reach a stabile level at around 6 hours. The two shortest classes (below 0.5 mm) increase their share of the length up to about 6 hours after which they remain stable. As expected little change in fibre length distribution is seen after 24 h for the two different controls.

**Figure 3:**
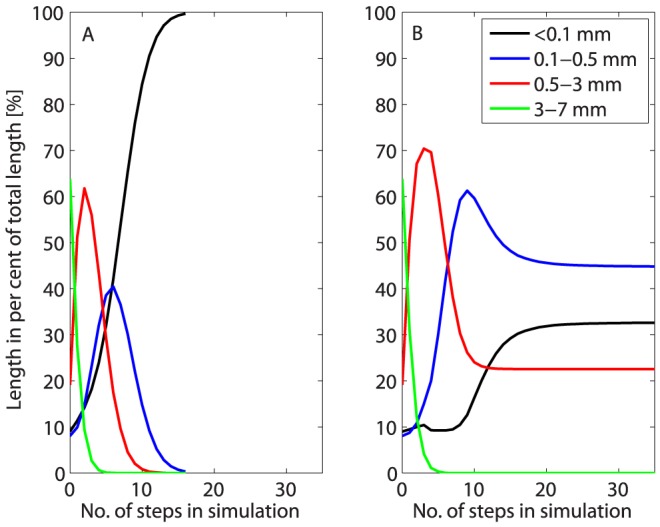
Two different simulated fibre length distributions for flax based on the actual fibre lengths prior to hydrolysis. A) random segmentation of fibres, B) segmentation according to the mechanical principles outlined in Figure 1 and equations (1) and (2). In the simulation in B), the parameter M_u_/P was set to 400 in step 1 and to decrease by 25% in each step until it reached 40.

Figure 3 shows results for two different simulations based on the actual fibre length distribution before hydrolysis (0 h in Figure 2). Figure 3a shows a simulation based simply on random segmentation of each fibre into two parts in each step, while Figure 3b shows a simulation based on the mechanical principles outlined in Figure 1 and equations (1) and (2). Both simulations show a gradual shortening of the fibres during the initial steps, but only the simulation based on mechanical principles (Figure 3b) correctly describes the stagnation in fibre lengths seen in the experimental data (Figure 2). It is no doubt possible to find alternative mathematical principles that would give the same stagnation as a model based on mechanical principles, but if only mathematically based, the model would merely be descriptive and would not offer a possible explanation to the observations. The simulation shown in Figure 3b captures the actual development well, but medium lengths are overestimated and short lengths underestimated during the first hours of the hydrolysis, presumably due to simplifications of the mechanical actions during free-fall mixing.

The enzymatic weakening of the fibre walls is implemented as a decrease in M_u_/P (Figure 1) during the simulation, i.e. a decrease in fibre strength, but fibres once discarded from the simulation because they are too short to break at the M_u_/P ratio used in that step are not included in later steps either. As the M_u_/P ratio decreases from step to step one could argue that this is not reasonable as discarded fibres will be eligible to attrition in a later step when the M_u_/P ratio has decreased sufficiently. However, this would translate into a situation where a constant low M_u_/P ratio was used throughout the simulation, and where all fibres would eventually end up in the shortest length class, which is not what is observed during hydrolysis (Figure 2). One may perhaps instead interpret the decrease in M_u_/P over time in the simulation as a way to *also* capture the variation in this parameter between individual fibres; strong fibres have a high M_u_/P and weak ones a low M_u_/P. This means that fibres discarded early during the simulation can be interpreted as strong fibres not subject to breakage, while the mean M_u_/P of the fibres remaining in the simulation decreases.

Elongated thick-walled plant cells such as fibres and tracheids have irregular regions within their cell walls, so-called dislocations or slip-planes, which have been found to be crack initiation points more often than the surrounding cell wall [15]–[17]. Segmentation of fibres at dislocations as a consequence of stirring combined with acid or enzymatic hydrolysis is a known phenomenon [12], [18]–[21]. That the simple simulation performed here seems to fit experimental data reasonably well do not conflict with this knowledge, as processed flax fibres like the ones used here are known to have numerous dislocations situated throughout their length [15], [22]. This implies that it is a justifiable simplification to assume that a fibre has the same material properties at all distances from the centre of the fibre, namely the properties of the irregular cell wall regions, as these are the main crack initiation points.

The simple simulation carried out in this work shows that particle size decrease during a biochemical process like enzymatic hydrolysis of plant biomass can be surprisingly easily described and explained if the mechanical side of the process is combined with the hydrolysis. As we see it this calls for the involvement of material science in biochemistry when processing of insoluble biomass is scaled from the lab to a full process.
